# ﻿A new species of *Bonnetia* Mart. (Bonnetiaceae) from the Pantepui of South America

**DOI:** 10.3897/phytokeys.247.126950

**Published:** 2024-10-08

**Authors:** Rafael G. Barbosa-Silva, Benjamin M. Torke, Pedro L. Viana

**Affiliations:** 1 Coordenação de Botânica, Museu Paraense Emilio Goeldi, Belém, Pará, 66077-830, Brazil Coordenação de Botânica, Museu Paraense Emilio Goeldi Belém Brazil; 2 Center for Biodiversity & Evolution, New York Botanical Garden, 2900 Southern Boulevard, Bronx, NY, 10458-5126, USA Center for Biodiversity & Evolution, New York Botanical Garden Bronx United States of America; 3 Instituto Nacional da Mata Atlântica, Santa Teresa, Espírito Santo, 29650-000, Brazil Instituto Nacional da Mata Atlântica Santa Teresa Brazil

**Keywords:** Amazonia, endemism, floristic inventory, Guayana Shield, herbarium, Malpighiales

## Abstract

*Bonnetia* is the most representative genus of the Pantepui woody flora and is among the groups with the greatest endemism in the local flora. The genus has 28 currently recognized species in tropical America, 26 of them endemic to the Pantepui. Here we describe *Bonnetiaayangannensis* from the summit of Mount Ayanganna tepui in Guyana, providing a morphological description, illustrations, distribution maps, characterization of micromorphology under scanning electron microscopy and leaf venation, comments comparing the new species with closely related species, and a key for the identification of the species of *Bonnetia* occurring in Guyana. With its restricted distribution threatened by climate change, *Bonnetiaayangannensis* is assessed in the conservation threat category of Critically Endangered. Its description raises the number of endemic species of *Bonnetia* in the Pantepui to 27.

## ﻿Introduction

*Bonnetia* Mart. is the largest genus of the Bonnetiaceae, comprising 28 species of trees and shrubs distributed across tropical America, with 26 species endemic to the Pantepui region of the Guayana Shield in northern South America (Weitzman et al. 2007). Species of *Bonnetia* often dominate woody landscapes of the tepui summits and upper slopes (Maguire 1972; Huber 1988, 2005, 2006; Barbosa-Silva et al. 2020), and the genus provides an excellent model for understanding vascular plant diversity and endemism within the Pantepui region (Riina et al. 2019). The seven species of *Bonnetia* occurring in Brazil were the focus of a recent taxonomic study (Barbosa-Silva 2020), but additional taxonomic work is needed, especially on the species in other parts of the Pantepui region.

Floral morphology holds significant value in the circumscription of genera of Bonnetiaceae. Within the genus *Bonnetia*, however, leaf venation patterns, particularly branching patterns, which are highly diverse in the genus, have been more important in the group’s taxonomy (Maguire 1972; Dickison and Weitzman 1996). Such characteristics have even been used to support generic segregates of *Bonnetia* (Maguire 1972; Steyermark 1984), but all these generic names are now treated as synonyms of *Bonnetia* (Barbosa-Silva 2020).

The most comprehensive advances in cataloging *Bonnetia* species diversity in the Pantepui resulted from several intensive floristic inventory programs in the region (see: Maguire 1972; Steyermark 1984; Barbosa-Silva et al. 2016; Kelloff et al. 2019). An example of these programs is the Smithsonian Institution’s Biological Diversity of the Guiana Shield (**BDG**) Program, which began in 1983 as an initiative aimed at studying, documenting, and preserving the biological diversity of the Guayana Shield over 30 years. Here, we describe a new species of the genus based on collections gathered by the BDG (Kelloff et al. 2019). Its description marks the first addition of a new species to the genus (and the family Bonnetiaceae) in 37 years (Sastre 1987). It raises to 27 the number of species of *Bonnetia* endemic to the Pantepui region, and to six the number of species occurring in Guyana.

## ﻿Materials and methods

All specimens of *Bonnetia* in the herbaria BM, COL, IAN, MG, MO, K, NY and US (acronyms follow Index Herbariorum, Thiers updated continuously) were examined. Specialized terminology for morphological structures follows Radford et al. (1974) and, for terms describing venation, Ellis et al. (2009). The description and illustration of reproductive structures was done from rehydrated material. Geographical data were sourced from specimen labels. The distribution map was generated using QGIS.org (2023). Assessment of the conservation status of the new species employed the categories and criteria of the IUCN Red List (IUCN 2012, 2024).

Samples of leaves, floral bracts, sepals and seeds from herbarium specimens deposited at NY were examined by Scanning Electron Microscopy (SEM), using a Hitachi model Su3500. Images were colored using Photoshop CS4 (Adobe). For the analysis of leaf venation, leaves were rendered transparent by soaking them in a 5% sodium hydroxide solution for two weeks at room temperature (Vasco et al. 2014). The transparent leaves were subsequently stained with 1% safranin and then immersed in ethanol to remove excess stain prior to imaging by an EPSON Perfection V750 PRO scanner.

## ﻿Results

### ﻿Taxonomic treatment

#### ﻿ Bonnetia ayangannensis

Taxon classificationPlantaeMalpighialesBonnetiaceae

Barb.Silva sp. nov.

BD5DADEC-043D-5C2F-8C7C-108727EF62C3

urn:lsid:ipni.org:names:77349807-1

##### Type.

**Figure 1.**
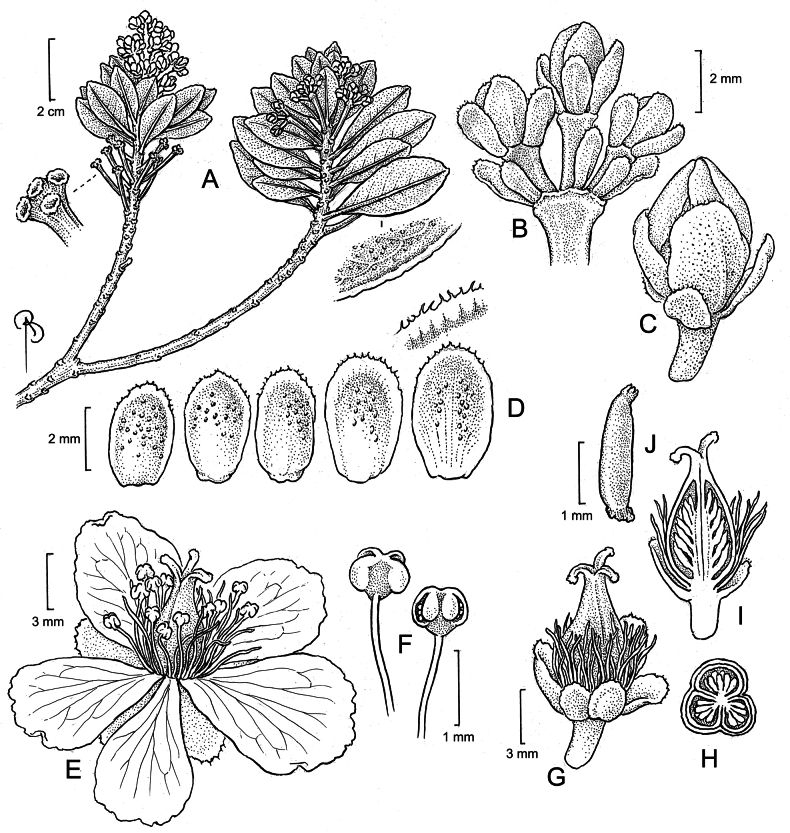
*Bonnetiaayangannensis***A** fertile branch, with details of the apex of a persistent inflorescence (at left) and the leaf abaxial surface and crenulate margin (at right) **B** immature inflorescence apex, showing bracts **C** flower bud **D** dimorphic sepals with glands, the detail showing the distal sepal margin **E** flower **F** stamens **G** closed capsule **H** transverse cut of the capsule **I** longitudinal cut of the capsule **J** seed (illustrated by: Bobbi Angell, from Radosavljevic et al. 247 and Clarke et al. 9524).

**Figure 2.**
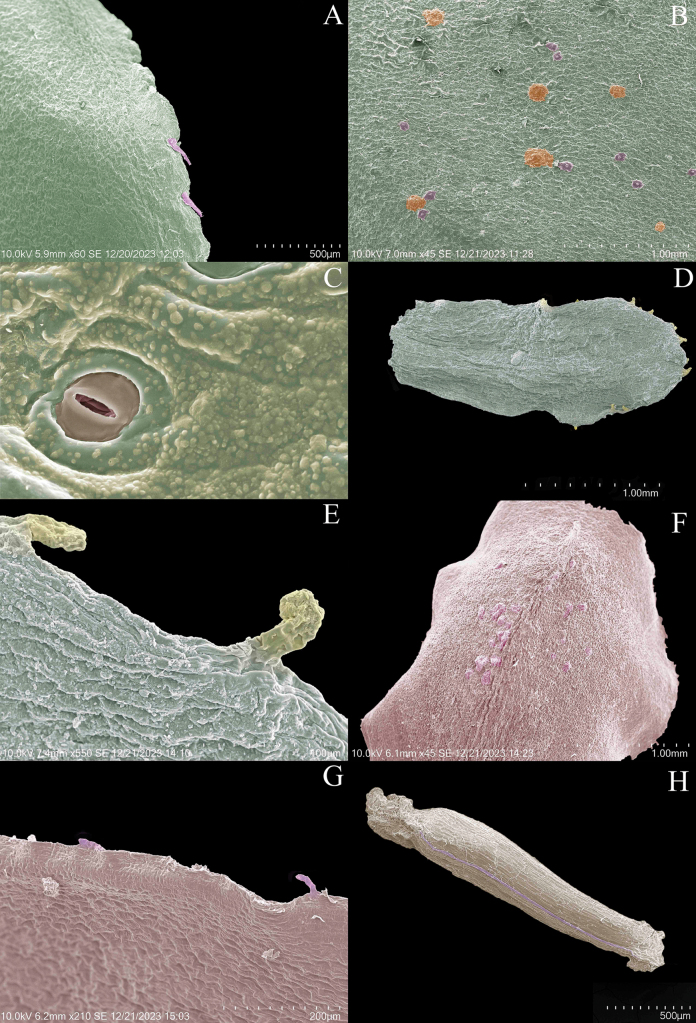
SEM images of the *Bonnetiaayangannensis***A** abaxial surface of the leaf showing the crenate margin, sometimes with deciduous spinulose projections (purple) **B** abaxial leaf surface with sessile glands (orange) and stomatal complexes (purple) at different levels **C** stomate immersed in a granular epidermis **D** bract **E** bract margin with short-stalked glands **F** sepals with sessile glands on the external surface **G** sepal margin with projections or short-stalked glands **H** seed (from Radosavljevic et al. 247 and Clarke et al. 9524).

**Figure 3.**
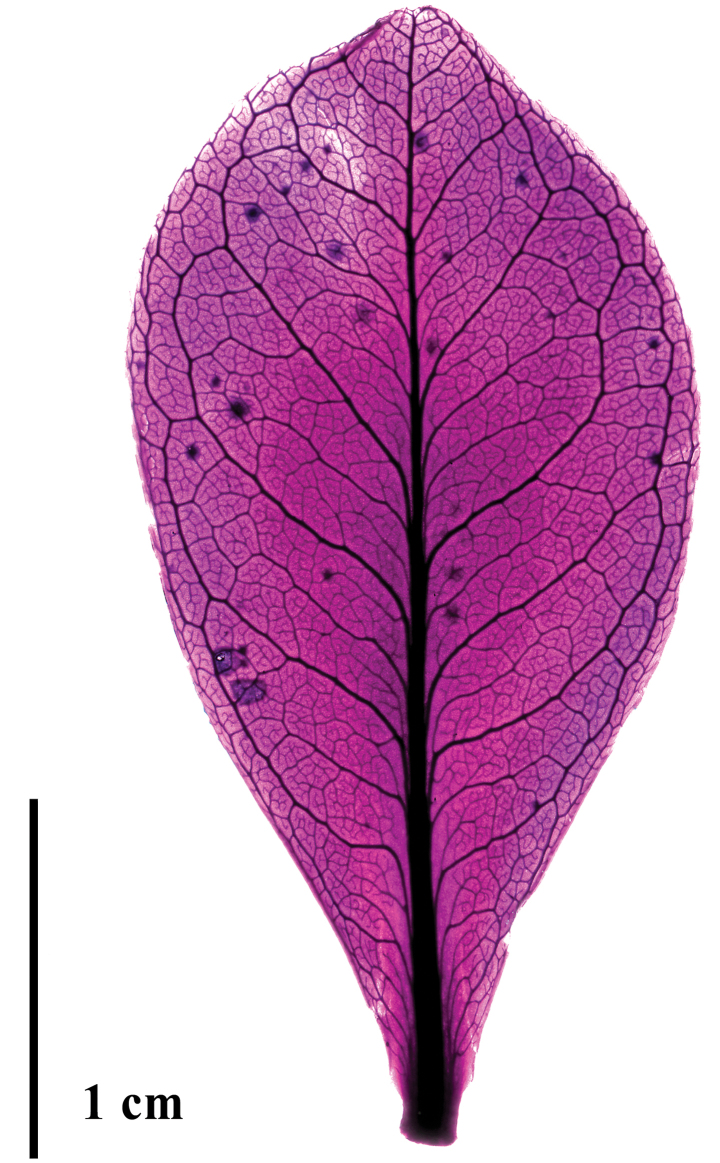
Leaf venation of *Bonnetiaayangannensis* (from Radosavljevic et al. 247).

**Figure 4.**
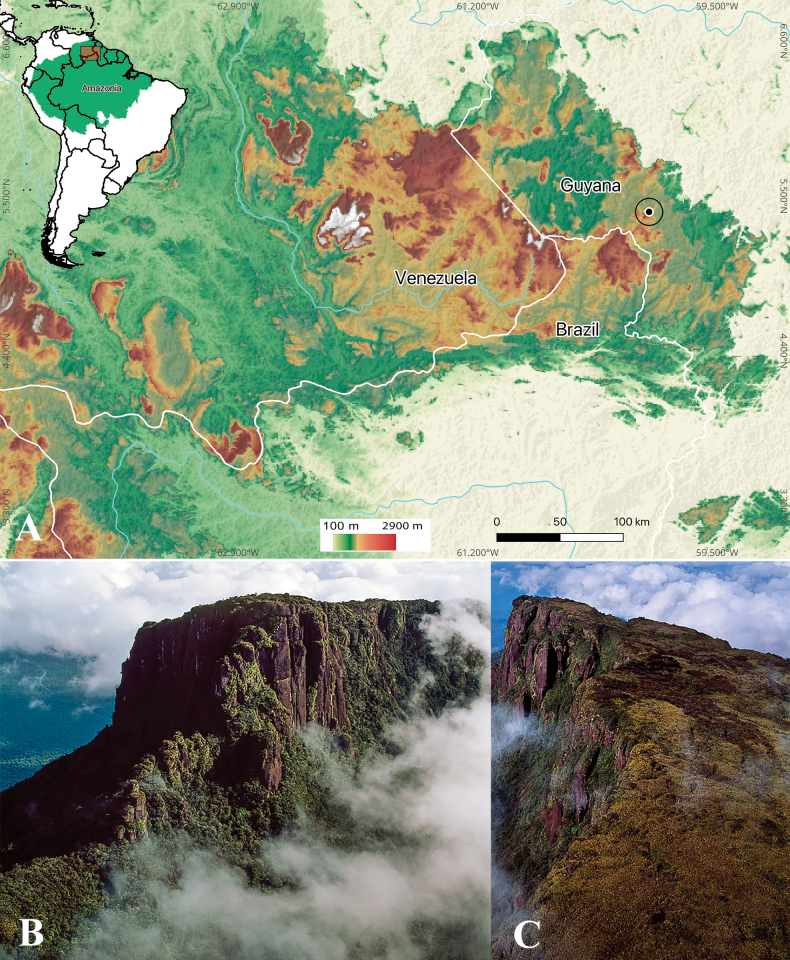
**A** The map shows a topographic representation where warm colors indicate higher elevations, illustrating the geographical distribution of *Bonnetiaayangannensis***B, C** view of Mount Ayanganna (photo by Adrian Warren/sasyimages.com).

Guyana • Potaro-Siparuni, Mt Ayanganna, East slope, summit plateau; 5°23′18"N, 59°58′54"W; 1955m; fl.; 20 Mar 2014; *A. Radosavljevic et al. 247* (holotype: NY 04078880, isotype US 03457967) (Figs 1–4).

##### Description.

Shrub or small tree up to 1.5 m tall. Branchlets often with remnants of inflorescences below the congested leaves. Leaves alternate, sessile or with a petiole c. 1 mm long, leaf blade (1–)1.5–4 × (0.5–)1–1.8 cm, coriaceous, obtrullate, rarely rhombic or narrowly rhombic, base cuneate, apex slightly rounded to acuminate, margins crenulate, abaxial surface glabrous, with sparse black glands when dried, secondary veins scarcely visible, adaxial surface glabrous, rarely with black glands; venation pinnate with no naked basal veins, one basal vein, and no agrophic veins (Fig. 4), the major secondaries semicraspedodromous with irregular spacing, the secondary angle slightly increasing proximally, and with decurrent attachment to the midvein, the minor secondaries and interior secondaries absent, the intersecondaries parallel, spanning less than 50% of the length of the subjacent secondaries, the intercostal tertiary veins irregularly reticulate, the epimedial tertiaries ramified with the admedial course parallel to the subjacent secondary and the exmedial course parallel to the intercostal tertiary, the exterior tertiaries terminating at the margin, the quaternary vein fabric irregularly reticulate, the quinternary vein fabric freely ramifying, areolation well developed, freely ending veinlets with two or more dendritic branches. Inflorescence axillary or occasionally terminal, once-paniculate, 2 cm long, with each branch bearing 3–4 flowers; bracts 2–3 × ca. 0.5 mm, oblanceolate to narrowly oblong, base truncate, rarely cuneate, apex rounded, margin ciliate; pedicels 2–3 mm long. Flowers 1.2–1.5 cm long, sepals 5, 2–4 × 1–2 mm, heteromorphic in size, oblong or ovate, apex rounded to acuminate, glabrous, margin ciliate, with sessile glands on the external surface; petals 5–6, 7–8 × 5–6 mm, broadly obtrullate, apex obcordate with asymmetric lobes, base cuneate, white with red margins; stamens 70–80, yellow, filaments 3 mm long, glabrous, anthers ca. 0.6 mm long; gynoecium ca. 5 × 2.5 mm, locules 3, carpels 3, green or yellow, stigma 3-branched in the upper fifth, the lobes reflexed. Capsules 7–8 × 3–3.5 mm, widely ovate to ovate. Seeds 1.5–2 mm long, linear.

##### Paratype.

Guyana • Potaro-Siparuni, Mt. Ayanganna, east face, edge of summit plateau; 5°23′18′′N, 59°58′56′′W; 2000 m elev.; fl., fr.; 24 June 2001; D. Clarke et al. 9524 (NY, US).

##### Notes.

*Bonnetiaayangannensis* is morphologically most similar to *B.paniculata* Spruce ex Benth., but it differs from that species by the leaf blades (1–)1.5–4 cm long (vs. 6–18 cm in *B.paniculata*), the leaves with glandular punctuations on the abaxial surface (vs. glands absent), the bracts 2–3 mm long (vs. 6–7 mm), the sepals ciliate and glandular (*vs.* cilia and glands lacking) and 2–4 × 1–2 mm (vs. 10 mm × 6–7 mm). *Bonnetiatepuiensis* Kobuski & Steyerm. and *B.rubicunda* (Sastre) A.L. Weitzman & P.F. Stevens also occurs on the summit of Mount Ayanganna, however the new species can be differentiated by the leaves cuneate at base (vs. rounded in *B.tepuiensis*), the flowers pedicellate (vs. sessile), arranged in inflorescences (vs. solitary flowers) and by having leaf with venation pinnate and flowers with sepals and petals less than 8 mm long (vs. parallel leaf venation and sepals and petals more than 18 mm long in *B.rubicunda*). For better identification of the *Bonnetia* species of Guyana, see the identification key below.

The voucher Clarke et al. 9535 (NY 04067354) is a mixed specimen, consisting of three branches of *B.tepuiensis*, and a small branch to the right of *B.ayangannensis*.

##### Etymology.

The epithet refers to the tepui Mount Ayanganna, where the new species was discovered.

##### Distribution and habitat.

*Bonnetiaayangannensis* appears to be endemic to the summit area of Mount Ayanganna tepui, Potaro-Siparuni, in western Guyana. The species is known from only two collections gathered in close proximity from a single population on the eastern summit slope of Mount Ayanganna. The species occurs in scrub forest on sandstone, together with *B.tepuiensis*, *Clusia* spp. (Clusiaceae), and *Brocchinia* spp. (Bomeliaceae), among others, at elevations between 1900 and 2000 m.

##### Preliminary conservation status.

We recommend that *Bonnetiaayangannensis* be assigned to the Critically Endangered category based on criterion B2ab(iv). The species is known from basically a single location (the two collection localities being separated by only c. 0.06 km), yielding an area of occupancy of 4 km^2^, assuming the default settings in GeoCAT (Bachman et al. 2011; Bachman and Moat 2012). However, considering that the area above 1900 m elevation on Mount Ayanganna, where taller forest gives way to scrub forest, is only about 0.7 km^2^, we suspect that the actual AOO is substantially less than 4 km^2^. Moreover, it is likely that the population will undergo anthropogenic climate change-driven reduction, paralleling reduction in the extent of the summit scrub vegetation on Mount Ayanganna, the summit peak of which is only 2041 m elevation. This projection is based on modeling (Nogué et al. 2009) that suggests upward migration and potential habitat loss for many species with restricted tepui distributions. This, in turn, subjects these localities to threats according to the conditions of subcriterion b(iv). Other species occurring in the eastern district of Pantepui, which are endemic to the summit of one or a few tepuis, are also threatened by rising temperatures, as is the case with *B.fasciculata* P.F.Stevens & A.L.Weitzman (Nogué et al. 2009) or are already listed on the IUCN Red List, such as *B.rubicunda* (Vulnerable) and *B.ptariensis* Steyerm. (Critically Endangered) (World Conservation Monitoring Centre 1998a, World Conservation Monitoring Centre 1998b).

##### Micromorphology.

The leaves have a crenulate margin, sometimes bearing deciduous spinular projections (Fig. 2A). The abaxial leaf surface is rugose (i.e., not smooth and presenting different levels) and bears many sessile glands and stomata (Fig. 2B). At higher magnification, the surface takes on a warty-crustose appearance with granular projections (Fig. 2C). The margins of the bracts have short stalked glands with thin-walled elongate heads (Fig. 2D, E). The sepals have sessile glands on the external surface (Fig. 2F) and short stalked glands on the margins (Fig. 2F, G). The seeds bear longitudinal striations (Fig. 2H).

### ﻿Key to the species of *Bonnetia* occurring in Guyana

**Table:** 

1a	Petals and capsules < 1 cm long	**2**
2a	Leaf blade rounded at base	***B.tepuiensis* Kobuski & Steyerm.**
2b	Leaf blade cuneate at base	**3**
3a	Flowers arranged in inflorescences	***B.ayangannensis* Barb.Silva**
3b	Flowers solitary	***B.roraimae* Oliv.**
1b	Petals and capsules > 1 cm long	**4**
4a	Flowers arranged in inflorescences	***B.paniculata* Spruce ex Benth.**
4b	Flowers solitary	**5**
5a	Leaf venation pinnate	***B.sessilis* Benth.**
5b	Leaf venation parallel	***B.rubicunda* (Sastre) A.L. Weitzman & P.F. Stevens**

## ﻿Discussion

Leaf characteristics hold significant taxonomic and historical value for the systematics of Bonnetiaceae. They provide support for the segregation of Bonnetiaceae as a distinct family (Dickison and Weitzman 1996) and have been used for the delimitation of genera now subsumed within *Bonnetia* (Maguire 1972). The micromorphological leaf features and leaf venation characters that we described for the new species may be of broader use in studies within the family, aiding in species delineation. For example, the warty-crustose leaf surface with a granular appearance that we observed in *B.ayangannensis* occurs in several other species of *Bonnetia*: *B.roraimae* Oliv., *B.steyermarkii* Kobuski, *B.wurdackii* Maguire (Dickison and Weitzman 1996). However, the spinulose projections on the leaf margin, which typically lack vascularization, are, as far as we know, shared only with *B.liesneri* Steyerm., although a similar structure is found in *B.ahogadoi* (Steyerm.) A.L. Weitzman & P.F. Stevens (Dickison and Weitzman 1996).

With respect to leaf venation, there is a fascinating array of branching patterns exhibited by different species of *Bonnetia*, ranging from pinnate to uniformly parallel (Maguire 1972; Dickison and Weitzman 1996). Our leaf clearing method showed that in *B.ayangannensis* the secondary veins are pinnate and the higher order veins, which in uncleared leaves are almost imperceptible, are irregularly reticulate, with the quaternary veins delimiting well defined areolae. Comprehensive phylogenetic sampling of *Bonnetia* is necessary to test whether species with pinnate (most species) and parallel veins (i.e., *B.fasciculata* A.L. Weitzman & P.F. Stevens, *B.maguireorum* Steyerm., *B.multinervia* (Maguire) Steyerm., and *B.rubicunda*) form distinct clades.

As is the case for *B.ayangannensis*, many plant species, including species of herbs, epiphytes, and trees, appear to be endemics of single mountains in the Pantepui region (Riina et al. 2019). The conservation of these species is of grave concern, in light of ongoing and projected impacts of climate change on the vegetation of the tepuis (Nogué et al. 2009; Vieira et al. 2024). These projections suggest reductions in the areas covered by unique summit vegetation types and upward migration of species. In many cases, these trends may result in the total loss of narrowly endemic species as current habitats become unsuitable for their occupation. With many species teetering on the brink of losing their climatic suitability, urgent conservation efforts are warranted to safeguard the biodiversity of these fragile ecosystems.

## Supplementary Material

XML Treatment for Bonnetia ayangannensis

## ﻿Additional information

### Author ORCIDs

Rafael G. Barbosa-Silva https://orcid.org/0000-0002-0668-4840

Benjamin M. Torke https://orcid.org/0000-0001-8823-3519

Pedro L. Viana https://orcid.org/0000-0001-5044-0758

### Data availability

All of the data that support the findings of this study are available in the main text.
